# Observations upon Some of the Medical Plants Mentioned by Shakspeare

**Published:** 1833-07

**Authors:** Samuel Rootsey

**Affiliations:** Corresponding Member of the Medico-Botanical Society.


TRANSACTIONS OF THE MEDICO-BOTANICAL SOCIETY OF LONDON.

*Published under the Direction of the President and Council.*

### *Observations upon some of the Medical Plants mentioned by Shakspeare.* Communicated by Samuel Rootsey, Esq., Corresponding Member of the Medico-Botanical Society.

*Hemlock.* I was lately inquiring the particular species which the Welsh call Cegyd, and was told it differed from hemlock. Hemlock, they said, grew in gardens, like parsley; but Cegyd grew in moist hedges, with a smooth spotted stalk. Shakspeare likewise speaks of hemlock as a corn-field plant, which can be no other than the Æthusa Cynapium.

> " Crown'd with rank fumiter and furrow weeds,
> With harlocks, *hemlock*, nettles, cuckow-flowers,
> Darnel, and all the idle weeds that grow
> In our sustaining corn."—*Lear*, act iv. sc. 4.

In another place he is very precise in distinguishing it from *kecksies*, by which name I have always heard the Conium maculatum distinguished in Essex.

> " Her fallow leas
> The darnel, *hemlock*, and rank fumitory
> Doth feed upon, while that the coulter rusts
> That should deracinate such savagery.

> The even mead, that erst brought sweetly forth
> The freckled cowslip, burnet, and green clover,
> Wanting the scythe, all uncorrected rank
> Conceives by idleness, and nothing teems
> But hateful docks, rough thistles, *kecksies*, burs,
> Losing both beauty and utility."—*K. Henry V.*, act v. sc. 2.

This word *kecksies* is evidently the Welsh Cegyd, and the Latin Cicuta.

It was the root of hemlock which was used as an ingredient in the poisonous cauldron of the Witches in *Macbeth*.

> "The root of *hemlock* digg'd in the dark."—Act iv. sc. 1.

As the Conium maculatum is likely to be meant in this place, I think the Æthusa should be called by Withering's name of *lesser hemlock*.

The etymology of the word hemlock is obscure. I consider that the word is derived from its ill smell, and consists of the aspirate H prefixed to the radix, which in Greek is Moly, from μολυνω, to *moil*, or defile. Hence it is properly applicable to the Allium Moly, and the Ligusticum Pelopponense, which latter I suppose to have been the Concion of the Greeks.

*Fumiter*, or *Fumitory*. This double orthography of our poet illustrates the etymology of this word. It takes its name of Fumus terræ from its almost aerial lightsomeness and glaucous colour.

*Thistle*. The word thistle seems to belong to the Dipsacus, called in English Teasle, and in Latin Carduus, from its having the shape of a heart. Wool is carded and teased by means of the Dipsacus fullonum. I suppose it takes the name of Dipsacus from its thirsty nature; for in all weathers it holds between its leaves an abundance of water.

*Harlock*, *Charlock*, *Scharlock*, and *Scarlet*, seem to be the same word with garlick, and perhaps carrot, and originally applicable to the Sinapis arvensis, or the Allium moly, or some other plant of a more orange colour. In this neighbourhood, the name of Carlock cups is given to the Ranunculi, and perhaps the Caltha; and, after all, the Calendula may be the true plant from whence the name of scarlet is derived.

*Nettles,* I have no doubt, receive their name from their use as a substitute for hemp in the construction of nets, and therefore the word applies to Urtica, although in this place it may be supposed as equally applicable to Lamium, or the dead nettle.

*Cuckoo-flowers*. This name is applied to three genera, Cardamine, Lychnis, and Orchis. On asking a poor woman for the name of the Lychnis diocia alba, she said it was called Ladies' attire:

> "And lady-smocks, all silver white,
> And cuckoo-buds of yellow hue."—*Love's Labour Lost,* act v. sc. 2.

a name certainly preferable to that of our poet for the Cardamine pratensis, to which he evidently applies it. The orchis here is called Ganderglasses and Gandergaws, but in Essex Cuckoo-flower. The woodseer, called in the New Forest Cuckoo-spit, and which, as I was informed in Sweden, the peasants there consider as the cause of madness in cattle, abounds upon the Lychnis and the Cardamine, and seems to indicate why they should bear the name of Cuckoo-flowers. Linnæus gives to the Ragged Robin the name of Flos cuculi, which I consider to be less entitled to it than the dioica; for I believe the latter to be Shakspeare's plant. The flowers of the Cardamine are considered to be antispasmodic; but, in the New Forest, the Genista anglica is administered to children for those convulsions that accompany dentition.

The cuckoo-*buds* of the above passage must be the Caltha palustris, and may possibly be our author's cuckoo-*flowers*.

*Darnel* is said to be a poison which destroys the sight: its quality is narcotic, and its name I suppose to be from the same radix as the Greek *δαρθανω, dormio*. The word ray, or in French *juray*, seems to be the Greek *αιρα*, and to mean poison, as *æs æris* in Latin, whence *ærugo*.

> "'Twas full of *darnel*, do you like the taste?"—1*st Part K. H. VI.* act iii. sc. 2.

*Burnet*, in Latin *sanguisorba*, so called from its use being chiefly confined to young females, appears to me to be equivalent to the Greek word *parthenium*, a name generally applied to another plant used as an emmenagogue.

*Cowslips*. The soporific principle of these flowers may perhaps reside in their freckles. The poet has pointed to it, in his *Midsummer Night's Dream*, with peculiar beauty and elegance:

> "The cowslips tall her pensioners be,
> In their gold coats spots you see;
> Those be rubies, fairy favours:
> In those freckles live their savours.
> I must go seek some dewdrops here,
> And hang a pearl in every cowslip's ear."—Act ii. sc. 1.

The agreeable odour of these flowers would indicate that the virtue, if extracted by the chemist, might combine the advantages of opium with those of saffron; and the profusion in which the plant is found renders it very deserving the attention of the physician.

*Clover*. This, I imagine, derives its name from its leaf being cloven: it would then have the same meaning as *clubs* at cards.

*Docks.* This herb may take its name from its penetrating into the ground. A *dog* may also be supposed to be named from the propensity of the terrier to *dig* into the earth. The genus Rumex, particularly the species Hydrolapathum, or Aquaticus, presents us with an excellent astringent. Its affinity to the rhubarb, and the use made of the species R. acutus, would entitle it to rank high in the materia medica. The Britannica of the ancients was our common water-dock, and I am not aware that we possess a more powerful or more eligible native astringent than this plant.

*Burs.* Woodville, in his Medical Botany, figures the Arctium lappa, which prefers a drier situation than the A. Bardana; the latter must therefore be considered the plant of Shakspeare. The name is doubtless of the same meaning as briar, and seems to imply that it is borne away by the passing traveller, to whose clothes the flowers or the stems strongly adhere.

*Darnel.* I might have remarked, under Darnel, that the Myrica gale seems to possess a similar intoxicating property, and is used sparingly for that purpose in Norway, in their drink. Being driven, by stress of weather, into Humbroe Sound, near Lillasand, I entered the house of the pilot, who had a great bunch hung up in his room. He spoke English, and, in answer to a question of mine, he informed me of its use there, and that its English name was *porse.*

*Mandrake.* There are two plants which are denominated Mandrake by our countrymen; they have large and forked roots. Of these, the Bryonia diocia is largest, white, and hairy; the Tamus communis is smaller, dark, and smooth. Shakspeare compares *Justice Shallow* to these roots.

"I do remember him at Clement's Inn, like a man made after supper of a cheese-paring. When he was naked, he was for all the world like a forked radish, with a head fantastically carved upon it with a knife; he was the very genius of famine, yet lascivious as a monkey, and they called him Mandrake." *King Henry IV.* act iii. sc. 2.

The etymology of the word is from its root being generally divided and forked, like a man.

"Semihominis mandragoræ flores."—*Colum.*

It is also denominated Mandragon by Gerard. The English word man exists in the Latin *humanus;* likewise in the Hebrew, and other languages. In the present instance, it is found in the Greek combined with the word *dracon,* from δερκω, *aspicio.*

Littleton supposes the word is substituted for *andragoras,*

from ανηρ, *vir,* and αγορεω, *loquor.* "Quod humanam speciem quodammodo etiam vocem quum evellitur, si vera tradunt referat ejulans?" To this Shakspeare adverts:

> "And shrieks like mandrakes torn out of the earth:
> That living mortals, hearing them, run mad."—*R. & Jul.* a. iv. s. 2.

Again,

> "Would curses kill as doth the mandrake's groan,
> I'd—" *2d Part K. Henry VI.* act iii. sc. 2.

From hence it appears that the plant was believed to utter a horrible and fatal shriek when dug out of the earth.

We receive a small forked root from Chinese Tartary, the name of which is Ginseng. I have always believed that the Chinese etymology of this word was *Jin-seang,* (*vide* Morr. 8868,) Mr. Morrison, however, in No. 8803, gives it differently; and in part iii. p. 187, he gives it thus, *jin-san,* from *jin,* a man, and *san,* gradual; its slow growth being supposed, according to him, to have suggested the name. I am still of opinion that the form 8803 is rather derived from 8868, or *jin-seang,* man's likeness; and I have no doubt but it has the same meaning as the word mandrake, although the origin of the term may have been obscured by its antiquity.

Mr. Morrison illustrates the use of this root in the following paragraph, which, as it indicates its virtue, I may be pardoned for transcribing. "The true Shang-tang gin-seng may be essayed by two men walking together a few miles, one having jin-san in his mouth, and the other with his mouth empty. When he who has nothing in his mouth is panting exceedingly, the other's breath will be just as usual." This passage reminded me of Shakspeare's

> "One poor pennyworth of *sugar-candy,* to make thee long-winded."

> *King Henry IV.* act iii. sc. 3.

Thus, ginseng appears to be chewed in the celestial empire as tobacco is with us. Loureiro shews that the Canadian root, Nin-sing, is very different in quality, as well as in appearance. Galen, writing of mandrake, observes, that its virtue resides in its rind, or bark, and he considers it as cold in the highest degree. By his terms, hot and cold, we must understand the acrid and the narcotic of modern toxicologists; and those plants the temperature of which, by the old writers, was considered as of the third and fourth degree, must merit our particular attention. The plant of Galen was the Atropa mandragora, and the Circæum of Pliny, employed by Circe in her incantations. Shakspeare alludes to it when, to imply madness, he says,

> "I think you all have drank of *Circe's cup.*"—*C. of Errors,* act v. sc. 1.

And again,

"Or have we eaten of the *insane-root*,
That takes the reason prisoner?—*Macbeth*, act i. sc. 3.

As the lurid Solanum Melongena, Melanzana, or Mala insana, *mad-apples*, is evidently named from its effect upon the brain, so the analogous root of the Atropa mandragora must be the insane-root of Shakspeare. It was also administered in the liquid form; for *Cleopatra* says,

"Ha, ha,
Give me to drink *mandragora*,
That I might sleep out this great gap of time."—*Ant. & Cl.* a. i. sc. 5.

The syrup of it was likewise given as the syrup of poppy:

"Not poppy nor mandragora,
Nor all the drowsy syrups of the world,
Shall ever medicine thee to that sweet sleep
Which thou hadst yesterday."—*Othello*, act iii. sc. 3.

As the hemlock and the mandrake, so the hebenon, &c. were directed to be gathered at midnight.*

"Thoughts black, hands up, drugs fit, and time agreeing,
Confederate season, else no creature seeing.
Thou mixture rank of *midnight weeds* collected,
With Hecate's ban thrice blasted, thrice infected,
Thy natural magic and dire property
On wholesome life usurp immediately."—Act iii. sc. 1.
(*Pours the poison into the sleeper's ears.*)

Being a native of the countries of Circe and Medea, it was no doubt one of those which the latter collected to renovate Æson.

"In such a night
Medea gathered the enchanted herbs
That did renew old Æson."

The Parisian Circea Lutetiana is by our botanists denominated *Enchanter's*, or Enchantress's nightshade; but I generally give it the shorter name of Hagwort, and, in the manner of Pythagoras, I dedicate it to the number 2, as the Horse-chestnut and Ragwort to the numbers 7 and 13, expressive of the major and minor modes in music, and of the weeks and lunations in astronomy.

The mandrake of Scripture, which had a remarkable smell, was evidently the flower of a different plant. In Hebrew it is *Dudain* or *Davidaim*, as it were Flos amoris, or Flor-amor; and hence probably our word Daffodils.

"That come before the swallow dares, and take
The winds of March with beauty."—*Winter's Tale*, act iv. sc. 3.

The daffodils of Milton were, however, our Crown imperials, as, lamenting the death of Lycidas, he says, alluding to the nectaries of that flower,

* Vide Hebenon, p. 22.

"Bid Amaranthus all his beauties shed,
And Daffodillies fill their cups with tears,
To strew the laureate hearse where Lycid lies."

*Poppy.* Having found that our Foxglove is denominated poppies in the New Forest, I have inquired in this neighbourhood also, and find that it bears the same name here; and sometimes it is contracted into *pops*. A reason is given, that the flowers can be popped upon the hand. The Papaver therefore is improperly called in English by that name. The seeds of the purple Papaver are known by the name of maw-seed, which indicates the proper English appellation of that plant; and this agrees with its name in the different languages that are dialects of the Teutonic, and also with the Greek *mecon*, &c. Shakspeare, however, when he alludes to its entering into the composition of drowsy syrups, must have meant the Papaver somniferum.

*Long purples.* The name of Foxglove, or Folk's-gloves, Finger-flower, or Digitalis, and Dogfingers, as it is called in Wales, together with the magnificent spike of purple flowers borne by the Digitalis purpurea, induce me to conjecture that this plant is alluded to by our illustrious poet as long purples:

"There is a willow grows ascant the brook,
That shews his hoar leaves in the glassy stream;
Therewith fantastic garlands did she make
Of crow-flowers, nettles, daisies, and long purples,
That liberal shepherds give a grosser name,
But our cold maids do *dead-men's fingers* call them."—*Hamlet*, iv. 7.

The common *blue-bells*, to which my late excellent friend, R. A. Salisbury, Esq. attached the epithet of *festalis*, might perhaps be thought to be the garland-flower of *Ophelia;* but Lightfoot says it is the Orchis mascula, though Martyn considers that the name of Dead-men's fingers would better apply to the palmated species. Lightfoot, thinking probably that he had discovered the liberal name, may have supposed, upon this foundation alone, that the plant was an orchis. What this liberal name is in reality may be known to gentle shepherds, but by me is only supposed to be the same which Dampier has applied to a South American tree, whose flowers may perhaps resemble those of our digitalis in form, if not in colour. In Hampshire, the Lotus corniculatus is called dead-men's fingers, but in the vicinity of Bristol the plant has various names; fingers and toes, devil's fingers, devil's claws, and crow-toes. The last seems to point it out as the tufted crow-toe of Milton's Lycidas. Gerard, however, in his Index, applies this name to the hyacinth, which, by Johnson, in his supplementary Appendix to his edition of Gerard, is called crow-leek.

Gerard gives the name of *Crow-flowers* to the Lychnis floscuculi, while to another species, the dioica, he has attached that of crow-soap, which latter, in Johnson's appendix, is made synonymous with Saponaria, or soapwort. I think none of these were the crow-flowers of the poet. The Caltha palustris is called by that name in this part of the country, and is much used by children in their garlands and festivities, together with the flowers of Ranunculus bulbosus and R. acris, which are called Craysies and Mayflowers. The latter term in Middlesex is given to the Iris palustris. In Essex, the flowers called May are those of the Prunus spinosa, rather than the Cratægus oxyacantha. This discrepancy in our English names may be considered as a reproach to science; but the botanist, who delights in the contemplation and study of wild words as well as wild flowers, may find an ample field, or rather garden, for his erudition, in comparing the synonymes of British and European plants, especially those whose faculties were discovered and appreciated by our experienced and benevolent ancestors, who extended their researches

"To every herb that sips the dew."

The Caltha I take to be the Mary-buds of Shakspeare.

"And winking mary-buds begin
To ope their golden eyes,
With everything that pretty bin,
My lady sweet arise."—*Cymbeline,* act ii. sc. 3.

The Marygold is the Calendula.

"The marygold that goes to bed with the sun,
And with him rises weeping."—*Winter's Tale,* act iv. sc. 3.

*Hebenon.* Shakspeare ascribes the death of *Hamlet* to the juice of hebenon having been poured into his ear. As he beautifully describes the action of the poison, I transcribe the entire passage.

*Ghost.* "Sleeping within mine orchard,
(My custom always of the afternoon,)
Upon my secure hour thy uncle stole,
With juice of cursed hebenon in a vial,
And in the porches of mine ears did pour
The leperous distilment; whose effect
Holds such an enmity with blood of man,
That, swift as quicksilver, it courses through
The natural gates and alleys of the body,
And, with a sudden vigour, it doth posset
And curd, like eager droppings into milk,
The thin and wholesome blood: so did it mine;
And a most instant tetter bark'd about,
Most lazar-like, with vile and loathsome crust,
All my smooth body."—*Hamlet,* act i. sc. 5.

The word hebenon means black, the *h* being a non-essential letter.* Hence I conceive this plant to have been the Atropa

* Vide Mandrake, p. 20.

Belladonna, which, where it is wild in Glocestershire, is by the country people called, from the colour of its fruit, Inkberries. From the following passage, I think it may have been used for poisoning darts and javelins:

> "Love's golden arrow at him should have fled,
> And not death's *ebon* dart, to strike him dead."—*Ven. and Adon.*

I make no doubt that the name of Henbane is a corruption of hebenon, and strictly applicable to the blackberry of the Dwale, so called from its effect in making us *dull* and sullen. (Hence Solanum? *Lethale.*)

> "And duller shouldst thou be than the fat weed
> That roots itself in ease on Lethe's wharf,
> Wouldst thou not stir in this."—*Hamlet*, act i. sc. 5.

As for the word *nightshade*, given probably from its forming a shed for the night, this seems appropriate to the dulcamara, whose bending twigs form an arbour like the clematis. I once imagined, though I have not had an opportunity of proving it, that the nightshade possessed the property of shedding and diffusing a peculiar smell during the night, and that it derived its name accordingly. This property has furnished appellations to the lily and the lilac. Whether the coagulations of the blood and the eruptions upon the skin, which attended the exhibition of hebenon, characterize the operation of belladonna, I must submit to the judgment of toxicologists, but I believe the description applies more strictly to that medicine than to any other.

---

TRANSACTIONS OF THE MEDICO-BOTANICAL SOCIETY OF LONDON.

*Published under the Direction of the President and Council.*

